# Macrophages facilitate post myocardial infarction arrhythmias: roles of gap junction and KCa3.1

**DOI:** 10.7150/thno.34801

**Published:** 2019-08-14

**Authors:** Yu-Dong Fei, Qian Wang, Jian-Wen Hou, Wei Li, Xing-Xing Cai, Yu-Li Yang, Liu-Hui Zhang, Zhi-Xing Wei, Tai-Zhong Chen, Yue-Peng Wang, Yi-Gang Li

**Affiliations:** 1 Department of Cardiology, XinHua Hospital Affiliated to Shanghai Jiao Tong University School of Medicine, Shanghai, China; 2 Department of Ophthalmology, XinHua Hospital Affiliated to Shanghai Jiao Tong University School of Medicine, Shanghai, China

**Keywords:** myocardial infarction, arrhythmias, macrophage, gap junction, KCa3.1

## Abstract

Effective therapeutic targets against post-myocardial infarction (MI) arrhythmias remain to be discovered. We aimed to investigate the role of macrophages in post-MI arrhythmias.

**Methods**: Mononuclear cell accumulation, macrophage polarization from M0 to M1 subset, and gap junction formation were analyzed in MI patients and MI mice by flow cytometry, immunofluorescence and patch clamping. Differentially expressed genes were identified by RNA sequencing. Macrophages and cardiomyocytes were cocultured in vitro, and the effects of gap junction and KCa3.1 on electrophysiological properties were assessed by patch clamping. The effects of KCa3.1 inhibition on post-MI arrhythmias were assessed by intracardiac stimulation and ambulatory electrocardiograms in vivo.

**Results**: Percentage of pro-inflammatory mononuclear cells were significantly elevated in patients with post-MI arrhythmias compared with MI patients without arrhythmias and healthy controls (p<0.001). Macrophages formed gap junction with cardiomyocytes in MI border zones of MI patient and mice, and pro-inflammatory macrophages were significantly increased 3 days post-MI (p<0.001). RNA sequencing identified *Kcnn4* as the most differentially expressed gene encoding ion channel, and the upregulation is mainly attributed to macrophage accumulation and polarization into pro-inflammatory subset. In vitro coculture experiments demonstrated that connection with M0 macrophages via gap junction slightly shortened the action potential durations (APDs) of cardiomyocytes. However, the APD90 of cardiomyocytes connected with M1 macrophages were significantly prolonged (p<0.001), which were effectively attenuated by gap junction inhibition (p=0.002), KCa3.1 inhibition (p=0.008), KCa3.1 silencing (p<0.001) and store-operated Ca^2+^ channel inhibition (p=0.005). In vivo results demonstrated that KCa3.1 inhibition significantly decreased the QTc durations (p=0.031), intracardiac stimulation-induced ventricular arrhythmia durations (p=0.050) and incidence of premature ventricular contractions (p=0.030) in MI mice.

**Conclusion**: Macrophage polarization leads to APD heterogeneity and post-MI arrhythmias via gap junction and KCa3.1 activation. The results provide evidences of a novel mechanism of post-MI heterogeneous repolarization and arrhythmias, rendering macrophages and KCa3.1 to be potential therapeutic targets.

## Introduction

Sudden cardiac death remains a worldwide critical health-related problem, and it is caused by ventricular arrhythmias secondary to acute myocardial infarction (MI) in approximately 80% of cases 1. Early clustering of ventricular tachycardia (VT) or ventricular fibrillation (VF) accounts for most of the mortality related to post-MI arrhythmias. Profound changes in cardiac electrophysiology occur in both ischemic and non-ischemic myocardium after acute coronary occlusion, leading to repolarization heterogeneity 2. As a result, ventricular arrhythmias could be initiated and sustained by multiple re-entrant circuits across the heterogeneous repolarization zones 2. Inflammatory macrophages might also play crucial roles in post-MI arrhythmias 3, but the mechanisms remain not fully understood.

Macrophages reside in all organs of vertebrates and perform distinct functions 4. It is demonstrated that resident macrophages could facilitate electrical conduction in the atrial ventricular node by forming gap junction with cardiomyocytes 5. Macrophages are well recognized to play critical roles in inflammation after MI 6, and the population of recruited macrophages quickly overwhelms the resident macrophages 7. Macrophages can be classified into different subtypes 8, and non-activated macrophages (M0 macrophages) can polarize into the classical pro-inflammatory macrophages (M1 macrophages), which dominated during 1-3 days post-MI 9. Till now, the effects of macrophages on cardiac electrophysiology during pathological conditions, especially on post-MI arrhythmias, remain poorly defined.

In the present study, we performed RNA sequencing between MI mice and sham operated mice, and *Kcnn4* was identified as the most differentially expressed gene encoding ion channel. *Kcnn4* encodes KCa3.1, the intermediate conductance calcium (Ca^2+^)-activated potassium channel. KCa3.1 is widely expressed in immune cells including macrophages, and previous studies demonstrated significant upregulation of KCa3.1 expression in M1 macrophages 10. KCa3.1 channel is voltage independent and Ca^2+^ sensitive, and its activation preserves the negative membrane potential required for sustained Ca^2+^ influx via store-operated Ca^2+^ (SOC) channels 11, which is the major way of Ca^2+^ entry in non-excitable cells. KCa3.1 has been reported to be a potential therapeutic target of catecholaminergic polymorphic ventricular tachycardia 12, but its role in post-MI arrhythmias remains not fully understood.

In our study, in vitro experiments revealed that M1 macrophages prolonged action potential durations (APDs) of cardiomyocytes via gap junction and KCa3.1 activation, and in vivo study showed that KCa3.1 inhibition attenuated post-MI arrhythmias. Taken together, our results demonstrated macrophages facilitate post-MI arrhythmias via gap junction and KCa3.1 activation, and KCa3.1 might be a promising therapeutic target against post-MI arrhythmias.

## Methods

All experiments were approved by the Ethics Committee of XinHua Hospital Affiliated to Shanghai Jiao Tong University School of Medicine. All animal procedures were conducted in accordance with the Guide for the Animal Care and Use Committee of XinHua Hospital and the guidance for the care and use of experimental animals published by NIH (the 8th Edition, NRC 2011).

### Clinical studies

All enrolled patients were informed with written consent prior to inclusion in the study. Human peripheral blood was collected from healthy volunteers (controls) and non-ST segment elevation MI patients without percutaneous coronary intervention at 3 days post-MI. The control group enrolled patients with normal coronary angiography results and no cardiac disease. MI was diagnosed according to the following three criteria of the World Health Organization: a typical history, electrocardiographic findings, and a significant increase in serum enzymes. Among the MI patients, those who survived ≥ 1 episodes of VT or VF were classified into the post-MI arrhythmias group, and the others were categorized into MI without arrhythmias group. Patients with significant concomitant diagnosis including malignancy, serious infections, autoimmune diseases or electrolyte disorders were excluded.

The left ventricular myocardial tissue sample was collected from the MI border zone of a patient who died from sudden death post-MI without percutaneous coronary intervention. The patient did not suffer from cancer, infectious or autoimmune diseases.

### Flow cytometry analysis

Peripheral blood mononuclear cells (PBMCs) were isolated from 2 ml heparin-treated blood by density gradient centrifugation using Ficoll Paque (GE Healthcare, Piscataway, NJ, USA). After blocking the Fcγ receptors, PE-conjugated anti-CD16 antibody (Biolegend) and APC-conjugated anti-CD14 antibody (Biolegend) were used for flow cytometry. After the PBMCs were incubated with antibodies, washed and fixed, the samples were analyzed by flow cytometry (BD Accuri C6 instrument, BD Biosciences, San Jose, CA, USA) and analyzed with FlowJo software (Tree Star).

M0 and M1 RAW264.7 macrophages were incubated with PE-conjugated anti-KCa3.1 antibody (Santa Cruz) to analyze the expression of KCa3.1, and were incubated with Fluo-3AM (Sigma) to detect intracellular Ca^2+^ by flow cytometry.

### Induction of MI in mice and administration of TRAM34

8-week-old male C57BL/6 mice (SLAC Laboratory Animals, China) were randomized to subject to permanent ligations of the left anterior descending branch of the coronary artery or to a sham operation without ligation, as described previously 13. In brief, mice were anesthetized by 2% isoflurane inhalation and mechanically ventilated, the thorax was opened via left thoracotomy to expose the heart, and the coronary artery was ligated with a 6-0 silk suture. Successful occlusion of the vessel was confirmed both by the presence of myocardial blanching in the perfusion bed and dynamic changes of electrocardiogram (ECG). Sham-operated animals underwent the same procedure without ligation. Mice died within 24 h after surgery were excluded from the experiment. Immediately after MI induction or sham operation, mice were randomized to receive TRAM34 (120mg/kg, Sigma) or same volume of vehicle (corn oil, Sigma) subcutaneously every day until sacrifice.

### RNA sequencing

RNA was extracted from ventricular tissues of MI border zones of MI mice and sham operated mice 3 days after surgery (n=3 in each group) using RNeasy isolation kit (Qiagen), and checked using a NanoPhotometer spectrophotometer (IMPLEN, CA, USA) and an Agilent 2100 Bioanalyzer (Agilent Technologies, CA, USA). Sequencing libraries were generated using VAHTSTM mRNA-seq V2 Library Prep Kit for Illumina, purified by AMPure XP system and assessed on the Agilent Bioanalyzer 2100 system. Paired-end sequencing of the library was performed on the HiSeq XTen sequencers (Illumina, San Diego, CA). The quality of sequenced data was evaluated by FastQC (version 0.11.2). RSeQC (version 2.6.1) was used for statistics of the alignment results. The homogeneity distribution and the genome structure were checked by Qualimap (version 2.2.1). Transcripts per million was computed by StringTie (version 1.3.3b) to compare gene expressions between samples to eliminate the influence of gene lengths and sequencing discrepancies. Q values were p values after multiple comparison adjustment. Genes with the q values lower than 0.05 and more than 2-fold increase or decrease were defined as differentially expressed genes.

### Cell isolation and culture

Primary fibroblasts and macrophages were isolated from adult mice as previously described 9. In brief, adult mice were deeply anesthetized with pentobarbital sodium (50 mg/kg, i.p.) and intracardially perfused with 40 mL of ice-cold PBS to exclude blood cells. The hearts were dissected and minced, followed by enzymatic digestion with a cocktail of collagenase (type II, Worthington, Lakewood, NJ), proteinase (Sigma) and DNase I (Sigma) for 60 minutes at 37 °C with gentle agitation. After digestion, the tissue was triturated and filtered through a 70-μm strainer to obtain cell suspension. To obtain fibroblasts, the cell suspension was centrifuged, and the cell pellets were re-suspended and incubated at 37 °C for 45 min. Cells attached to the bottom of dishes were taken as cardiac fibroblasts. To obtain macrophages, leukocyte-enriched fractions were isolated from the cell suspension by Ficoll Paque (GE Healthcare, Piscataway, NJ, USA), and macrophages were purified by magnetic activated cell sorting positive selection using anti-F4/80 magnetic microbeads (Miltenyi Biotec) according to manufacturer's instructions.

Primary cardiomyocytes from adult mice were isolated using Langendorff apparatus. Briefly, mice hearts were rapidly removed and cannulated and perfused with Ca^2+^ free Tyrode solution containing collagenase (type II, Worthington, Lakewood, NJ) and proteinase (Sigma). The standard Tyrode solution contains (in mM): 135 NaCl, 5.4 KCl, 1.0 MgCl_2_, 0.33 NaH_2_PO_4_, 10 Glucose, 10 HEPES and 1.8 CaCl_2_, pH was adjusted to 7.4 with NaOH. After perfusing for 30 min at 37°C, the hearts were swirled in a culture dish, and cardiomyocytes were collected.

Neonatal mice ventricular cardiomyocytes (NMVMs) were isolated from the heart of 1- to 2-day-old neonatal C57BL/6 mice as previously described 14, and were maintained in Dulbecco modified Eagle medium (DMEM) supplemented with 10% fetal bovine serum (FBS).

RAW264.7 cell line was purchased from American Type Culture Collection (Manassas, VA). To induce M1 polarization, macrophages were incubated with 100ng/mL IFN-γ (Biolegend) and 10 ng/mL lipopolysaccharide (LPS) (Sigma) for 24 hours. For co-culture, 13 mm coverslips (VWR) were pre-seeded with (1-5)x10^4^ cells/cm^2^ RAW264.7 cells or RAW264.7 cells pretreated with LPS and IFN-γ, and NMVMs were seeded at a density of (0.2-1)x10^5^ cells/cm^2^ onto the coverslips. Medium were exchanged every other day, and patch clamp experiments were performed on the 3rd day. For hypoxia experiments, cells were cultured with FBS free medium in an incubator with 1% O_2_.

### Small interfering RNA (siRNA) targeting mice KCa3.1

The siRNA targeting mice KCa3.1 (5'-GCACCUUUCAGACACACUU-3') were synthesized by GenePharma (Shanghai, China). M1 macrophages were transfected with either siRNA targeting KCa3.1 or negative control using Lipofectamine 2000 (Invitrogen) according to the manufacturer's instruction.

### Patch clamping

All experiments were performed at 35 ℃ to 37 ℃. All cells were subjected to hypoxia for 24 hours before patch clamping. All reagents were purchased from Sigma unless otherwise indicated. Cells were patch-clamped using the whole-cell configuration as previously described 15, 16. For action potential recordings, patch pipettes (resistance 2 to 3 MΩ) were filled with pipette solution containing (in mM): 110 K-aspartate, 30 KCl, 5 NaCl, 0.1 EGTA,10 HEPES, 5 MgATP, 5 creatine phosphate, 0.05 cAMP, pH 7.2 with KOH. Lucifer yellow (0.5%) was added to pipette solution to indicate interconnected cells via gap junction. The shutter of fluorescence was opened for a few seconds at 1st minute, 3rd minute and 20th minute after cell membrane rupture to minimize photo toxicity. RMPs (resting membrane potentials) were obtained by gap-free mode. GAP26 (100μM), TRAM34 (1μM) or SKF96365 (10μM) were added to the bath superfusate. Action potentials were elicited with 2 ms duration and 2 × diastolic threshold current pulses at 1 Hz. After action potential recordings, the pipettes were carefully removed, and lucifer yellow gradually faded. To mark the exact patch-clamped cells, the coverslips were engraved with a glass marking diamond, as described by Salvarani et al 17. Subsequently the coverslips were picked up from the chamber for immunostaining.

For KCa3.1 recordings, pipette solution contained (in mM): 145 K-aspartate, 2 MgCl_2_, 10 HEPES, 10 K_2_EGTA, and 8.5 CaCl_2_ (1 μM free Ca^2+^), pH 7.2 with KOH. The external solution contained (in mM): 160 Na-aspartate, 4.5 KCl, 2 CaCl_2_, 1 MgCl_2_, and 5 HEPES, pH 7.4 with NaOH. KCa3.1 current was elicited with voltage ramps from -120 mV to +40 mV for 200 ms applied every 10 seconds, and KCa3.1 conductance was calculated from the slope of the current-voltage relationship at -80 mV.

Voltage and current signals were averaged and measured with a MultiClamp 700B patch-clamp amplifier (Axon Instruments, Sunnyvale, CA, USA) controlled by a personal computer using a Digidata 1440A acquisition board driven by pCLAMP 10 software (Molecular Devices, Sunnyvale, CA, USA).

### Histological analysis and immunofluorescence

The mice were deep anesthetized at the 3rd day after MI or sham surgery. The hearts were quickly removed and fixed in 4% paraformaldehyde, embedded in paraffin and sectioned at 5 microns. Cultured cells were fixed with 4% paraformaldehyde for 15 min, permeabilized with 0.2 % Triton X-100 for 20 min, blocked with 3 % BSA for 60 min at room temperature. Ventricular sections and cells were incubated with primary antibodies against α-actinin (Abcam), F4/80 (Abcam), iNOS (Abcam), connexin43 (Cx43) (Abcam), KCa3.1 (Alomone labs), CD163 (Abcam), CD68 (Abcam) and vimentin (Abcam), and subsequently with fluorescent secondary antibodies Alexa Fluor 488 (Invitrogen), Alexa Fluor 555 (Invitrogen) or Alexa Fluor 647 (Invitrogen). Cells were stained with DAPI for nuclei, and analyzed by confocal microscope (Leica) or scanner (3D Histech), and analyzed by Image-Pro Plus 6.0 software (Media Cybernetics) or Caseviewer software (3D Histech). More than 3 sections of ventricles from different mouse samples were randomly selected to quantify the number of macrophages.

### Western blotting

Mouse hearts were collected 3 days post-MI. Heart tissues or cells were solubilized in SDS-sample buffer, sonicated, subjected to SDS-PAGE and transferred to PVDF membranes. The membranes were incubated overnight at 4 °C with the primary antibody against KCa3.1 (Alomone labs) or GAPDH (Sigma) following the instructions. After thorough wash, the membranes were incubated with secondary antibody for 2 hours at room temperature, and signals were revealed by enhanced chemiluminescence ECL (Thermo Scientific) on an image-capturer (Tanon 5200) and quantified by Image J software (NIH).

### Ambulatory ECG telemetry and intracardiac programmed electrical stimulation

The transmitters of continuous ambulatory ECG telemetry (Ensense Biomedical Technologies, China) were fixed on the back of mice during anesthesia with isoflurane, and the leads were tunneled subcutaneously to the upper right and lower left chest in the lead II position. Telemetry data were recorded continuously via a receiver and PowerLab station (AD Instruments) placed near the mice. The ECG channels were filtered between 0.3 and 1000 Hz. Continuous ECG waves for more than 10 seconds were analyzed using LabChart software to obtain heart rates and QT intervals. QTc was calculated by Mitchelle formula 18. Occurrences of premature ventricular contractions (PVCs) within 60 minutes were counted by two researchers independently.

The inducibility of VT/VF was assessed by in vivo intracardiac programmed electrical stimulation on mice receiving TRAM34 or vehicle 3 days post-MI. A 1.1-F octapolar electrophysiological catheter (Millar Inc, Houston, TX, USA) was inserted into the right ventricular apex via the right jugular vein 3 days post-MI. For burst pacing, a pacing train of 30 stimuli (S1) was delivered at cycle lengths of 30 ms. For extra-stimulus pacing, 3 premature stimuli were delivered following 7 paced beats at a basal cycle length of 100ms.

### Data analysis

Results are presented as means ± standard error of means unless otherwise indicated. Data were analyzed by pCLAMP 10.1, Origin 8, GraphPad Prism 5 and SPSS 19.0 software. Statistical significance was assessed using two-tailed Student's t tests between two groups or one way analysis of variance with Bonferroni post hoc analysis among multiple groups. P < 0.05 was considered statistically significant.

## Results

### Pro-inflammatory mononuclear cells were increased in patients with post-MI arrhythmias

**Figure 1:**
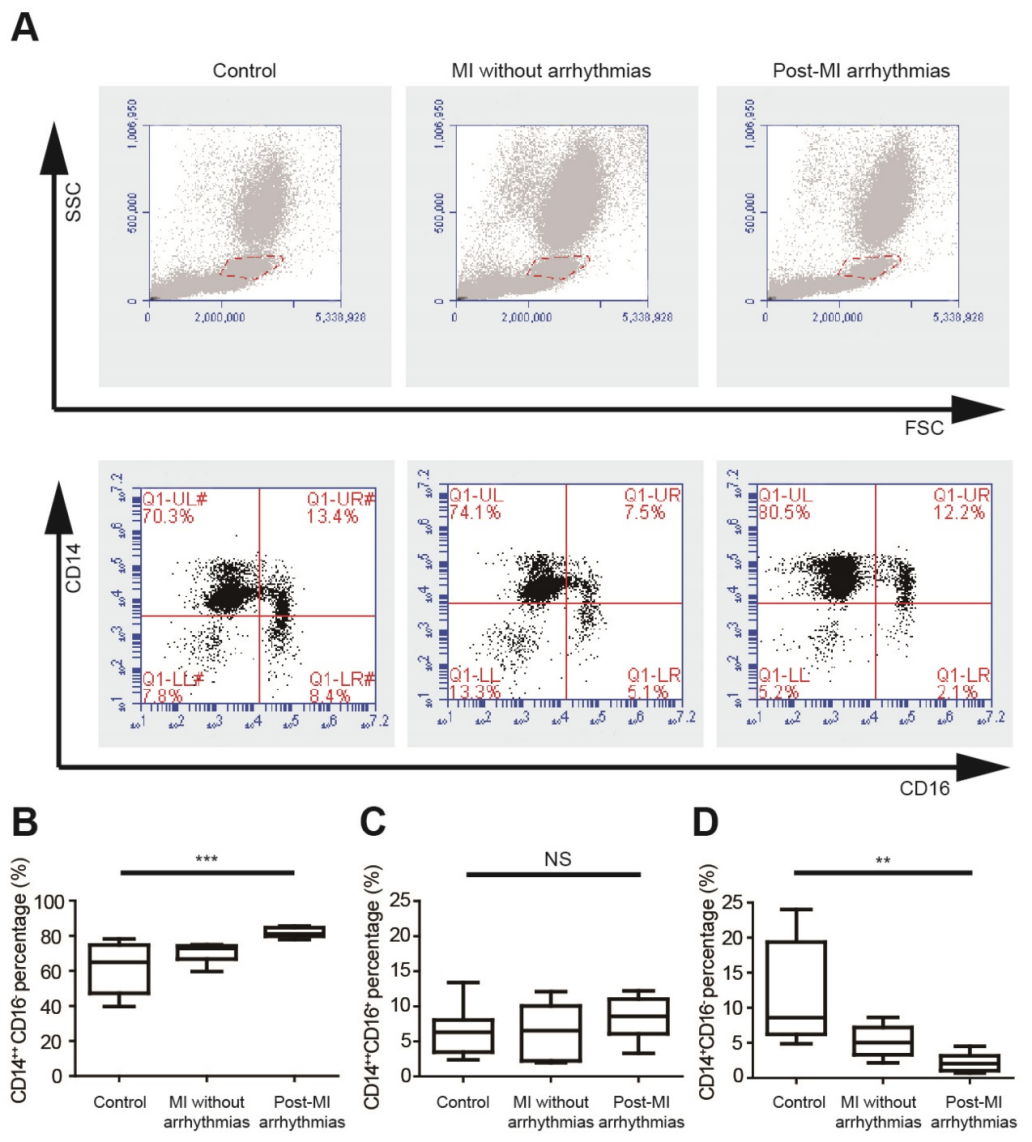
Pro-inflammatory mononuclear cells are increased in patients with post-MI arrhythmias at 3 days post-MI. (A). Gating strategy for analyzing monocyte subsets. FSC/SSC was firstly applied to distinguish monocytes from neutrophils and lymphocytes. Monocyte subsets were determined by CD14 and CD16 expression. (B-D). Statistical analysis of monocyte subsets in controls, MI patients without arrhythmias and patients with post-MI arrhythmias (n=8 in each group). **p<0.01. *** p<0.001. NS, not significant.

We analyzed the percentages of different monocyte subsets using flow cytometry in healthy controls, MI patients without arrhythmias and patients with post-MI ventricular arrhythmias (n=8 in each group) (Figure 1A, Table S1), and the left ventricular ejection fractions were not different between the latter two groups. Monocytes can be classified into pro-inflammatory CD14^++^CD16^-^ monocytes, intermediate CD14^++^CD16^+^ monocytes and non-classical CD14^+^CD16^+^ monocytes. Percentages of three monocyte subgroups in PBMCs were summarized in Figures 1B to 1D and Table S1. The percentage of CD14^++^CD16^-^ cells is significantly higher in patients with post-MI arrhythmias (81.84 ± 0.97%) than MI patients without arrhythmias (70.18 ± 1.88%, p=0.047) and controls (61.76± 5.00%, p<0.001). Percentages of CD14^++^CD16^+^ monocyte subset tended to be higher in patients with post-MI arrhythmias than those without post-MI arrhythmias (8.31 ± 1.04% vs 6.31 ± 1.38%, p=0.782). Compared with controls, percentage of CD14^+^CD16^+^ monocyte was lower in patients with post-MI arrhythmias (p=0.001) and in MI patients without arrhythmias (p=0.041).

### Macrophages formed gap junction with cardiomyocytes and accumulated after MI

**Figure 2:**
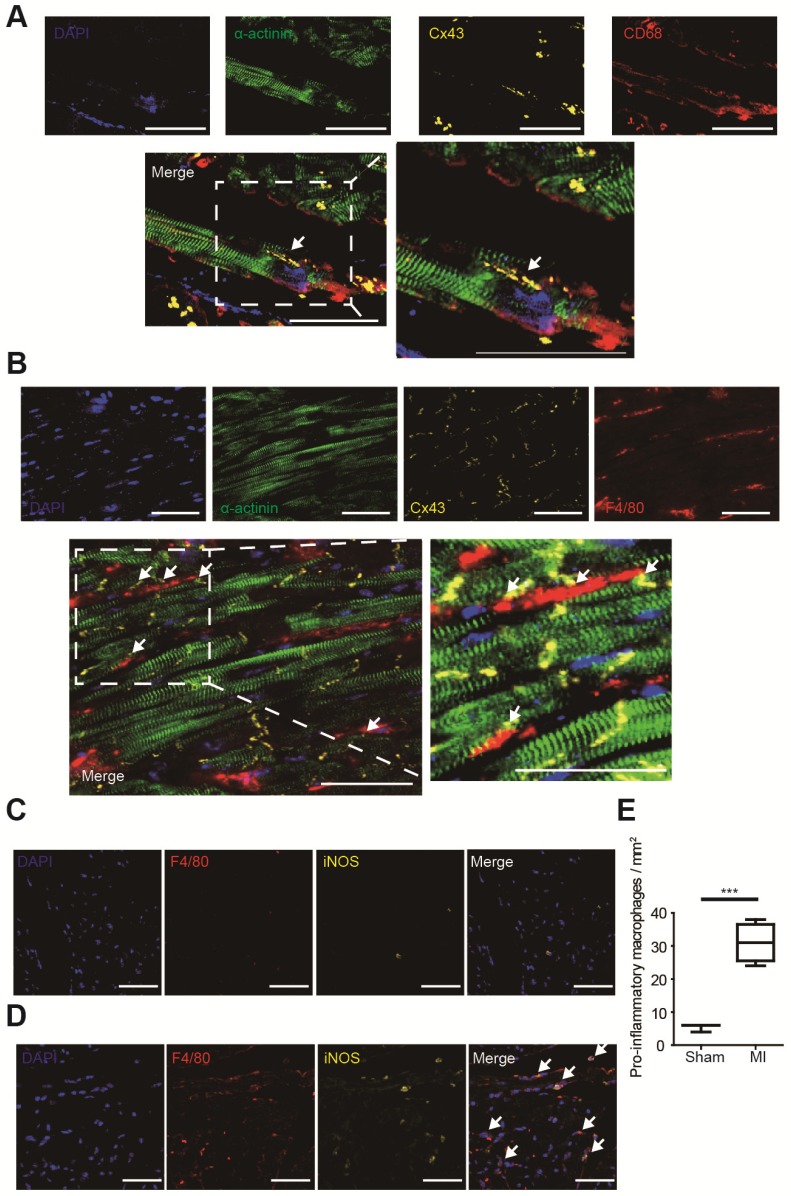
Macrophages form gap junction with cardiomyocytes and accumulate in MI border zones 3 days post-MI. (A-B). Triple immunofluorescence (cardiomyocytes = green, macrophages = red, Cx43 = yellow) in MI border zones of MI patient (A) and MI mouse (B). Arrows indicate gap junction between cardiomyocytes and macrophages. (C-D). Double immunofluorescence of F4/80 (red) and iNOS (yellow) in MI border zone of sham operated (C) and MI mice (D). Arrows indicate pro-inflammatory macrophages. (E). Statistical analysis of the numbers of pro-inflammatory macrophages in sham operated (n=3) and MI (n=4) mice. Scale bars indicate 50 μm. *** p<0.001.

Gap junction formation between macrophages and cardiomyocytes was analyzed by immunofluorescence in MI border zones of both human (Figure 2A) and mouse (Figure 2B). It is demonstrated that gap junctions between macrophages and cardiomyocytes are mainly formed by Cx43 5, and our triple immunostaining detected Cx43 between cardiomyocytes and macrophages. Triple immunostaining of sham operated mouse ventricles detected Cx43 between cardiomyocytes rather than between cardiomyocytes and macrophages (Figure S1A), because the density of macrophage is very low in normal ventricles (Figure 2C) in accordance with previous reports 5.

Macrophage accumulation in MI border zones was also evaluated. Few macrophages (F4/80^+^) were identified in sham operated mouse ventricles (Figure 2C). However, a markedly accumulation of macrophages, especially pro-inflammatory (F4/80^+^ iNOS^+^) macrophages was observed in MI border zones 3 days post-MI (Figure 2D). Macrophage densities were significantly elevated in MI mice (n=4, p<0.001, Figure 2E). Meanwhile, immunostaining results showed that few alternative (F4/80^+^ CD163^+^) macrophages were observed 3 days post-MI (Figure S2A).

### KCa3.1 was the most differentially expressed ion channel

**Figure 3:**
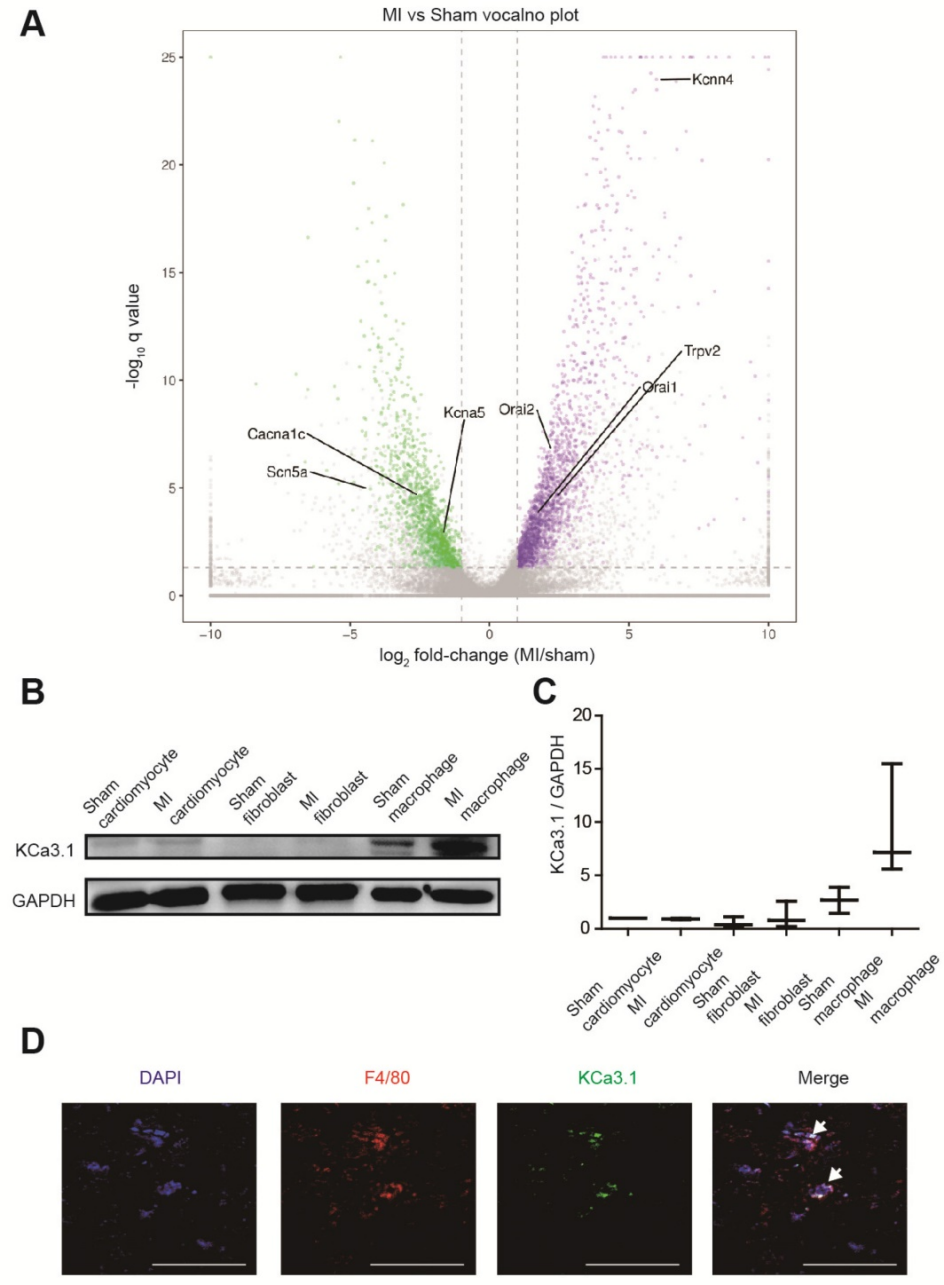
KCa3.1 is the most differentially expressed ion channel between MI and sham operated mouse hearts and it is mainly attributed to macrophage accumulation and polarization. (A). The volcano plot of differentially expressed genes between MI and sham operated mice by RNA sequencing (n=3 in each group). Samples were collected 3 days post-MI. The X-axis is the log_2_ fold-change (MI/sham), the Y-axis is -log_10_ q value (MI/sham). Up-regulated and down-regulated genes are shown in purple and green, respectively. Several differentially expressed genes encoding ion channels are annotated. (B-C). Western blotting results of KCa3.1 expression in different cells 3 days post-MI (B) and statistical summary (C) (n=3 in each group). (D). Double immunofluorescence of F4/80 (red) and KCa3.1 (green) in MI border zone of MI mice 3 days post-MI. Arrows indicate overlay of F4/80 and KCa3.1. Scale bars indicate 50 μm.

RNA sequencing identified more than 3000 differentially expressed genes between border zones MI mice and sham operated mice (n=3 in each group), and we paid close attention to genes encoding ion channels (Figure 3A, Table S2). *Kcnn4* is the most differentially expressed gene encoding ion channel, and it was up-regulated for more than 60 folds in MI mice. Then we assessed which cell contributed to the elevation of KCa3.1. Western blotting demonstrated that KCa3.1 was mainly expressed in macrophages rather than cardiomyocytes or fibroblasts, and the KCa3.1 expression was markedly higher in macrophages from MI hearts (Figures 3B and 3C). Immunofluorescence results confirmed that KCa3.1 overlaid with macrophage marker F4/80 (Figure 3D), but not cardiomyocyte marker α-actinin (Figure S2B) or fibroblast marker vimentin (Figure S2C).

### KCa3.1 current and expression were elevated in M1 macrophages

**Figure 4:**
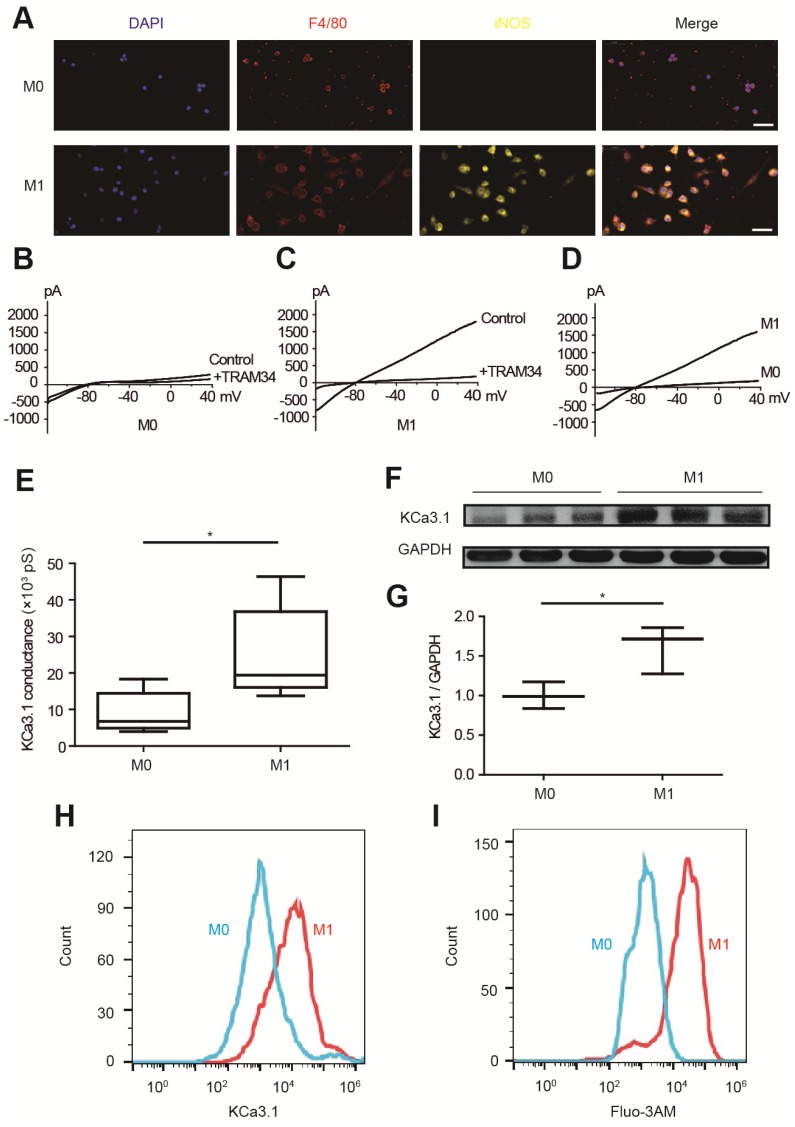
KCa3.1 and intracellular Ca^2+^ are elevated in M1 macrophages. (A). Double immunofluorescence of F4/80 (red) and iNOS (yellow) of M0 and M1 macrophages. (B-C). Representative curves of KCa3.1 before and after TRAM34 perfusion in M0 (B) and M1 (C) macrophages. (D-E). Subtraction of TRAM34 sensitive currents of M0 and M1 macrophages (D) and statistical analysis (n=6 in each group) (E). (F-G). Western blotting results of KCa3.1 expression of M0 and M1 macrophages (F) and statistical analysis (n=3 in each group) (G). (H). Flow cytometry analysis of KCa3.1 expression of M0 and M1 macrophages. (I). Flow cytometry analysis after application of Fluo-3AM to detect intracellular Ca^2+^. Scale bars indicate 50 μm. * p<0.05.

KCa3.1 current and KCa3.1 protein expression were analyzed by patch clamping, Western blotting and flow cytometry in RAW264.7 cells. M0 macrophages are round-shaped cells expressing F4/80 but not iNOS, while M1 macrophages are irregular-shaped and they express both F4/80 and iNOS (Figure 4A). KCa3.1 current was analyzed by whole-cell patch clamping. The same protocol was performed before and after TRAM34 perfusion (Figures 4B and 4C), and the subtraction results were calculated as KCa3.1 (Figure 4D). KCa3.1 conductance was significantly increased in M1 macrophages ((24.87±5.14) ×10^3^ pS, n=6) compared to M0 macrophages ((9.00±2.28) ×10^3^ pS, n=6, p=0.018) (Figure 4E). KCa3.1 protein expression was also significantly elevated in M1 macrophages analyzed by Western blotting (n=3, p=0.037, Figures 4F and 4G) and flow cytometry (Figure 4H). As KCa3.1 facilitates Ca^2+^ influx, we loaded macrophages with Ca^2+^-sensitive fluorophore Fluo-3AM. Flow cytometry demonstrated M1 macrophages showed higher fluorescence level, demonstrating they loaded higher intracellular Ca^2+^ (Figure 4I). RMPs were also measured, and RMPs of M1 macrophages (-54.38±1.75 mV, n=6) were not statistically different with M0 macrophages (-58.84±1.38 mV, n=6, p=0.073).

### Solitary NMVMs and NMVMs connected with macrophages were identified

Gap junctions between NMVMs and macrophages were confirmed by both lucifer yellow transfer and immunostaining. Lucifer yellow is able to transfer via gap junctions but not cell membranes 19. NMVMs were patch-clamped, and the clamped cells were confirmed to be NMVMs both by elicitation of typical action potentials and expression of α-actinin assessed by immunostaining later. Solitary NMVMs cocultured with macrophages were confirmed both by no adjacent cells under bright field and by no lucifer yellow transfer under fluorescence. If other adjacent cells besides the clamped NMVMs became fluorescent after membrane rupture, the NMVMs were identified as NMVMs connected with macrophages (Figure S3A). The subset of macrophage was determined by cell morphology and immunofluorescence results (Figure S3B).

### M0 and M1 macrophages electrically modulated cardiomyocytes oppositely via gap junction

**Figure 5:**
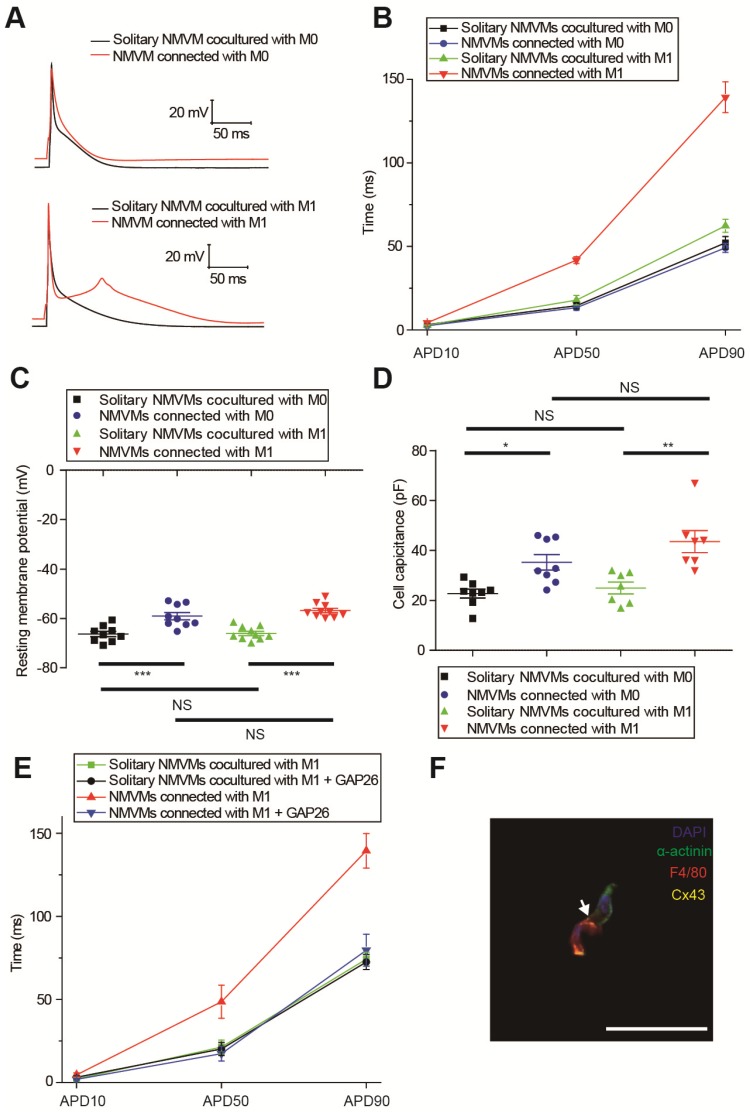
M0 and M1 macrophages electrically modulate cardiomyocytes oppositely via gap junction. (A-B) Representative action potential curves (A) and statistical summary (B) of NMVMs cocultured with M0 (n=14 for solitary NMVMs and connected NMVMs) or M1 macrophages (n=15 for solitary NMVMs and connected NMVMs). (C). Resting membrane potentials of NMVMs cocultured with M0 (n=9 for solitary NMVMs and connected NMVMs) or M1 macrophages (n=10 for solitary NMVMs and connected NMVMs). (D). Cell capacitances of NMVMs cocultured with M0 (n=8 for solitary NMVMs and connected NMVMs) or M1 macrophages (n=7 for solitary NMVMs and connected NMVMs) (E). Summary of APDs of solitary NMVMs cocultured with M1 (n=6) and NMVMs connected with M1 (n=8) before and after GAP26 perfusion. (F). Triple immunofluorescence of cocultured cells (cardiomyocyte = green, macrophage = red, Cx43 = yellow). Arrow indicate Cx43 between cardiomyocyte and macrophage. Scale bars indicate 50 μm.*p<0.05. **p<0.01. *** p<0.001. NS, not significant.

We compared the APDs, RMPs and cell capacitances between solitary cardiomyocytes and cardiomyocytes connected with macrophages in the same dish to exclude the effects of cytokines and medium. Representative APs were shown in Figure 5A, and APDs at 10% (APD10), 50% (APD50) and 90% (APD90) repolarization were quantified respectively (Figure 5B, Table 1). Compared with solitary NMVMs cocultured with M0 macrophages, connection with M0 macrophages slightly decreased APD10 (p=0.381), APD50 (p=0.647) and APD90 (p=0.535, n=14) of NMVMs. However, connection with M1 macrophages markedly increased APD10 (p=0.085), APD50 (p<0.001) and APD90 (p<0.001, n=15) of NMVMs in comparison with solitary NMVMs cocultured with M1 macrophages. 2 of 15 NMVMs connected with M1 macrophages displayed early afterdepolarization pattern during phase 2 of AP, as shown in Figure 5A.

Elevation of RMPs and cell capacitances also confirmed electrical coupling between cardiomyocytes and macrophages via gap junction. RMPs of cardiomyocytes were both elevated when connected with M0 (n=9, p<0.001) and M1 macrophages (n=10, p<0.001) (p<0.001, Figure 5C, Table 1), and the RMPs of NMVMs connected with M0 and NMVMs connected with M1 macrophages were not different (p=0.881). Cell capacitances were also significantly increased when connected with M0 (n=8, p=0.030) and M1 macrophages (n=7, p=0.001) (p<0.001, Figure 5D, Table 1). No statistical significance was found between cell capacitances of NMVMs connected with M0 and NMVMs connected with M1 macrophages (p=0.368).

To further confirm it is gap junction formation that led to the APD prolongation, we applied the Cx43-mimetic peptide GAP26 (100μM) to block gap junction (Figure 5E). While GAP26 did not exert obvious effects on APD90 of solitary NMVMs cocultured with M1 macrophages (n=6, p=0.790), application of GAP26 significantly reversed the prolongation of APD50 (p=0.017) and APD90 (p=0.002) of NMVMs connected with M1 macrophages (n=6, Table S3). We also performed triple immunostaining of α-actinin, F4/80 and Cx43 of cocultured cells, and the results further confirmed that cardiomyocytes and macrophages formed gap junctions (Figure 5F).

### KCa3.1 and intracellular Ca^2+^ elevation of macrophages played crucial roles in APD prolongation of cardiomyocytes

**Figure 6:**
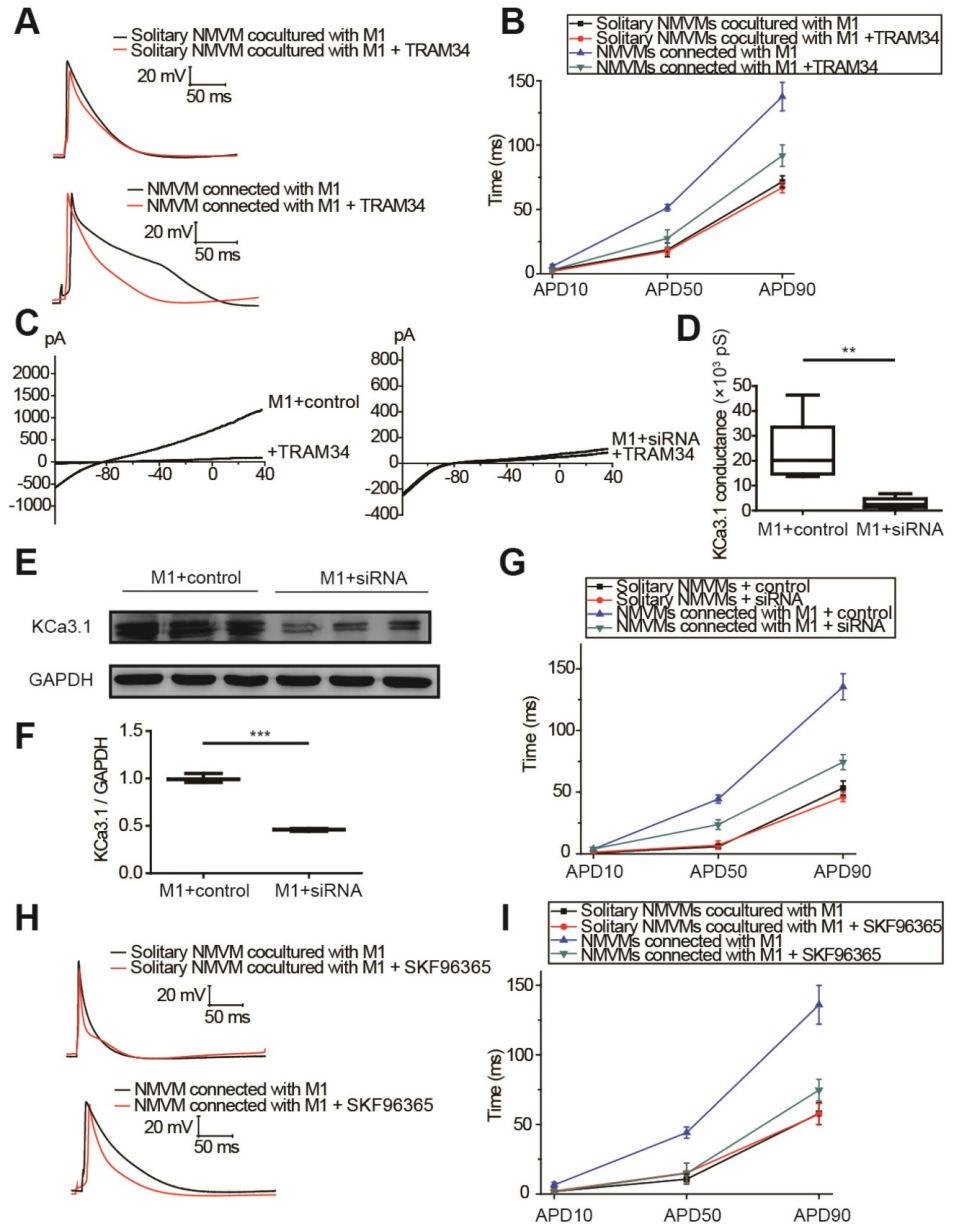
KCa3.1 and elevated intracellular Ca^2+^ of macrophages play crucial roles in APD prolongation of cardiomyocytes. (A-B). Representative action potential curves (A) and statistical summary (B) of solitary NMVMs cocultured with M1 (n=6) and NMVMs connected with M1 (n=6) before and after TRAM34 application. (C-D). KCa3.1 current curves (C) and summary (D) of M1 macrophages treated with siRNA (n=5) or negative control (n=5). (E-F). Western blotting results and statistical analysis of KCa3.1 expression of M1 macrophages treated with siRNA or negative control. (G). APDs of NMVMs connected with M1 after application of siRNA (n=6) or negative control (n=6). (H-I). Representative action potential curves (H) and summary (I) of solitary NMVMs cocultured with M1 (n=5) and NMVMs connected with M1 (n=5) before and after SKF96365 perfusion. **p<0.01. *** p<0.001.

The results of tissue RNA sequencing and cell expression analysis indicated KCa3.1 as a potential target (Figure 3), so we next investigated the effect of KCa3.1 on the APDs of cardiomyocytes. KCa3.1 specific inhibitor TRAM34 did not exert a difference in the APDs of NMVMs cultured alone (n=5, Figure S4). For cocultured cells, representative APs before and after TRAM34 application were displayed in Figure 6A, APD10, APD50 and APD90 were summarized in Figure 6B. TRAM34 did not modify the APDs of solitary NMVMs cocultured with M1 macrophages (n=6, Table S4). However, TRAM34 significantly attenuated the prolonged APD50 (p=0.014) and APD90 (p=0.008) of NMVMs connected with M1 macrophages (n=6, Table S4). RMPs of NMVMs connected with M1 macrophages were slightly elevated after TRAM34 (from -59.85 ±1.31 mV to -57.77 ±1.17 mV, n=6, p=0.264).

Next, we used siRNA against KCa3.1 to further prove the role of KCa3.1. Patch clamping verified that KCa3.1 conductance was markedly decreased from (23.32±5.92) ×10^3^ pS (n=5) to (2.93±1.01) ×10^3^ pS after siRNA treatment (n=5, p=0.009, Figures 6C and 6D). Western blotting demonstrated that siRNA markedly decreased the expression of KCa3.1 in M1 macrophages (n=3, p<0.001, Figures 6E and 6F). Application of KCa3.1 siRNA effectively reversed the prolonged APDs of NMVMs connected with M1 macrophages (n=6, APD10 p=0.930; APD50 p=0.003; APD90 p<0.001, Table S5) (Figure 6G).

As KCa3.1 channel facilitates Ca^2+^ influx via SOC channel in non-excitable cells including macrophages, we also analyzed the effects of SOC by applying the SOC channel blocker SKF-96365. Figures 6H and 6I showed that SKF-96365 significantly reversed the prolonged APD50 (p=0.008) and APD90 (p=0.005) of NMVMs connected with M1 macrophages (n=5, Table S6).

### Inhibition of KCa3.1 effectively attenuated post-MI arrhythmias in vivo

**Figure 7:**
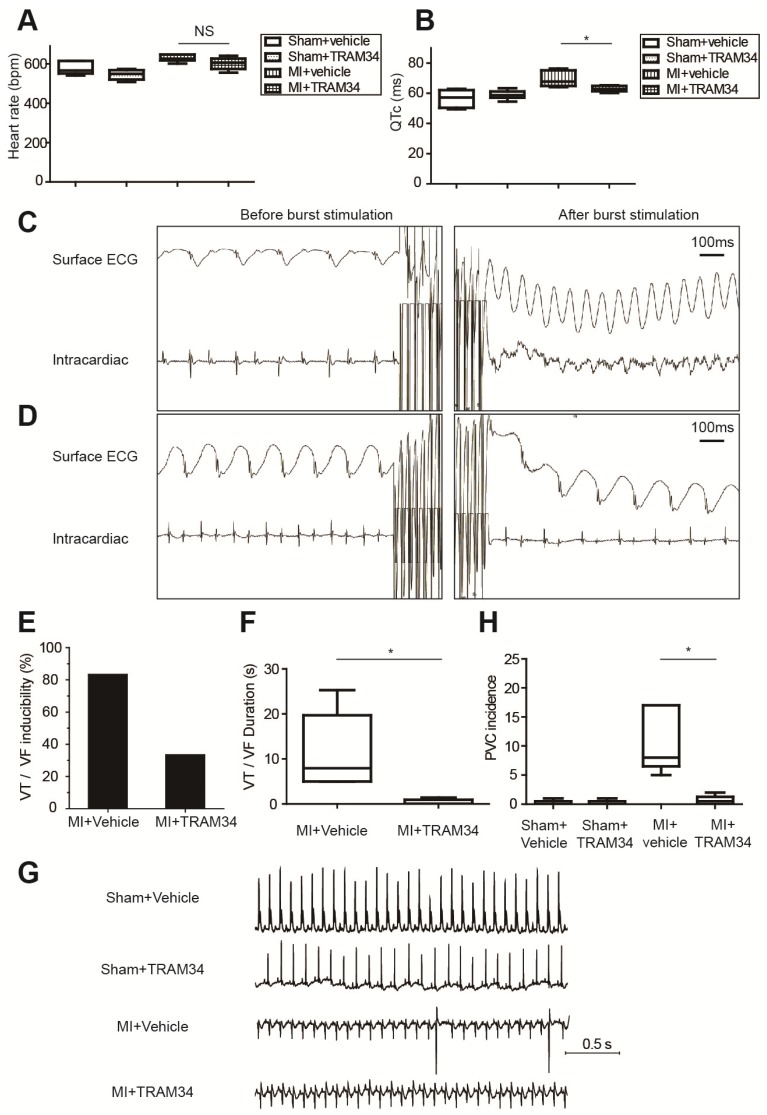
KCa3.1 inhibition effectively attenuated post-MI arrhythmias in vivo at 3 days post-MI. (A-B). Heart rates (A) and QTc durations (B) of sham operated mice treated with vehicle (n=5) or TRAM34 (n=6) and MI mice treated with vehicle (n=6) or TRAM34 (n=5). (C-D). Representative surface ECGs and images of intracardiac electrical stimulation of MI mice treated with vehicle (C) or TRAM34 (D). (E-F). VT/VF inducibility (E) and duration (F) of MI mice treated with vehicle (n=6) or TRAM34 (n=6). (G). Representative ambulatory ECGs of MI or sham operated mice treated with TRAM34 or vehicle. (H). Summary of PVC incidence in 60 minutes in MI or sham operated mice treated with TRAM34 or vehicle (n=6 in each group). * p<0.05. NS, not significant.

Ambulatory ECG data of MI or sham operated mice receiving TRAM34 or vehicle were analyzed respectively (n=5 or 6 in each group, Figures 7A and 7B, Table S7). TRAM34 did not significantly affect the heart rates of MI mice (p=0.128), but effectively decreased the QTc durations of MI mice (p=0.032, Figures S5A and S5B).

Representative intracardiac programmed electrical stimulation results of MI mice treated with vehicle or TRAM34 were shown in Figures 7C and 7D, respectively. TRAM34 effectively reduced the inducibility (Figure 7E) and the duration of VT/VF by burst pacing (n=6 in each group, p=0.05, Figure 7F) in MI mice. Post-MI ventricular arrhythmias were also induced by extra-stimulus pacing in vehicle treated MI mice (Figure S5C), and TRAM34 effectively prevented the arrhythmias (Figure S5D). Representative ambulatory ECG images were shown in Figure 7G. Few PVCs occurred in sham + vehicle and sham + TRAM34 group. Occurrences of PVCs were markedly increased in MI + vehicle group (12.33±4.60, n=6), and TRAM34 significantly attenuated post-MI PVCs (0.67±0.33, n=6, p=0.030, Figure 7H).

Moreover, application of TRAM34 for 3 days did not alter the accumulation of pro-inflammatory macrophages in MI border zones (Figures S6A and S6B, n=4, p=0.758). TRAM34 neither increased the number of alternative macrophages (Figures S6C and S6D, n=4, p=0.852) nor fibroblasts (Figure S6E) in MI border zones on the 3rd day post-MI. Also, administration of TRAM34 for 3 days did not significantly inhibit macrophage migration as analyzed by macrophage density within the infarct region (Figures S7A and S7B, n=4, p=0.143), and did not attenuate myocardial inflammation of the infarct zone (Figure S7C).

## Discussion

In the present study, we demonstrated that macrophage accumulation and polarization after MI led to repolarization heterogeneity and post-MI arrhythmias via gap junction and KCa3.1 activation. Flow cytometry results revealed that pro-inflammatory mononuclear cells were significantly increased in patients with post-MI arrhythmias. In MI border tissues, gap junctions were identified between macrophages and cardiomyocytes, and macrophages polarized to pro-inflammatory subtype 3 days post-MI. Macrophages and cardiomyocytes were cocultured in vitro. While connection with M0 macrophages slightly shortened the APDs of NMVMs, connection with M1 macrophages markedly prolonged the APDs of NMVMs. KCa3.1 activation in M1 macrophages facilitated Ca^2+^ influx, which can transfer through gap junction, leading to APD prolongation of cardiomyocytes. Gap junction inhibition, KCa3.1 inhibition or silencing, and SOC channel inhibition effectively attenuated APD prolongation respectively. Consistent with in vitro results, in vivo KCa3.1 inhibition significantly attenuated post-MI QTc prolongation and arrhythmias. These findings demonstrated the novel mechanism that macrophages facilitate post-MI arrhythmias, and the potential of KCa3.1 to be a therapeutic target.

### A novel mechanism of post-MI repolarization heterogeneity and arrhythmias

In our study, we demonstrated that different subsets of macrophages exerted completely opposite effects on APDs of cardiomyocytes via gap junction. While connection with M0 macrophages slightly decreased APD90 (from 52.14±3.81 to 49.19±2.74 ms, p=0.535), connection with M1 macrophages markedly increased APD90 more than twice (from 62.35±3.89 to 139.31±9.20 ms, p<0.001) (Figures 5A and 5B). Inhibition of KCa3.1 also decreased QTc of MI mice in vivo. Heterogeneous repolarization has been recognized as one of the main mechanisms of MI-induced arrhythmias. Previous studies attributed repolarization heterogeneity to increased extracellular potassium, Ca^2+^ transient alternans and different conduction velocities 2. Our results provided a novel mechanism of heterogeneous repolarization after MI. Under physiological conditions, the resident macrophages are M0 macrophages 5. However, after coronary artery occlusion, dramatic changes take place, immune cells accumulate in the border zone of infarction, macrophages polarize into pro-inflammatory subset, which dominate the population of macrophages during 1-3 days post-MI (Figures 2D and 2E) 9. Our results provided evidences that cardiomyocytes form gap junction with different subtypes of macrophages after MI, which results in APD variation, repolarization heterogeneity, and eventually post-MI ventricular arrhythmias. Ion channels (especially KCa3.1) of macrophages could be promising therapeutic targets against post-MI arrhythmias.

### Gap junction detection and confirmation

Gap junctions permit the direct passage of ions (electrical coupling) and small molecules up to 1000 Da (metabolic coupling) between interconnected cells. In our experiments, we applied both immunostaining and patch clamping to confirm the gap junction formation between cardiomyocytes and macrophages. Gap junctions between macrophages and cardiomyocytes are mainly formed by Cx43 5, and our triple immunofluorescence results detected Cx43 staining between cardiomyocytes and macrophages in human (Figure 2A) and mice (Figure 2B) MI border tissues and cocultured cells (Figure 5F). Patch clamping results confirmed both metabolic coupling (lucifer yellow transfer, Figure S3A) and electrical coupling (elevation of RMPs and cell capacitances, Figures 5C and 5D). Lucifer yellow (approximately 500 Da) is an established gap-junction permeable dye. For patch pipettes it is sufficient to obtain excellent cell staining by diffusion in approximately 3 minutes, and it does not change the electrophysiological properties of the cells 19. We observed for 20 minutes and even longer to exclude the possibility of its transfer through cell membranes. Lucifer yellow tends to fade even in the dark over time 19, so it will not interfere with the multi-color immunofluorescence results, and we adopted the method reported by Salvarani et al to mark the sites to find the exact cells 17. Our results also showed that cardiomyocytes with lucifer yellow diffused to M0 or M1 macrophages displayed more positive RMPs (Figure 5C) and larger membrane capacitances (Figure 5D) compared with solitary cardiomyocytes cocultured with macrophages, which demonstrated electrical coupling. Taken together, these results provided firm evidences that the cardiomyocytes and macrophages formed gap junctions.

Macrophages and cardiomyocytes are able to affect each other by indirect effects including cytokines 20, 21. In our experiments, we compared the electrical properties between solitary cardiomyocytes cocultured with macrophages and cardiomyocytes connected with macrophages in the same culturing environment, thus we were able to exclude the influence of cytokines and medium.

### KCa3.1 as a novel therapeutic target against post-MI arrhythmias

In our study, RNA sequencing identified *Kcnn4* as the most differentially expressed gene encoding ion channel between MI and sham operated mice (Figure 3A, Table S2). KCa3.1, encoded by *Kcnn4*, is significantly elevated in M1 macrophages (Figure 4). KCa3.1 inhibition or silencing significantly reversed the effect of M1 macrophages on APDs of cardiomyocytes in vitro (Figure 6) and attenuated post-MI arrhythmias in vivo (Figure 7).

Our in vivo results demonstrated that TRAM34 shortened QTc durations in MI mice (p=0.031, Figure 7B), which is in accordance with the in vitro results that TRAM34 attenuated the APD prolongation of cardiomyocytes connected with M1 macrophages. KCa3.1 blockers were demonstrated to decrease the spontaneous beating rate of sinoatrial node cells and reduce the arrhythmias in mice models of catecholaminergic polymorphic ventricular tachycardia 12. In our study, the effects of TRAM34 on heart rates in MI mice were not obvious compared to vehicle group (p=0.128). However, TRAM34 significantly decreased the QTc durations of MI mice. Therefore, it is reasonable that the antiarrhythmic effects of TRAM34 were due to attenuation of heterogeneous repolarization and prolonged QTc rather than decreasing heart rates.

In our study, TRAM34 was applied in vivo for only 3 days, and immunostaining results showed that pro-inflammatory macrophages (Figures S6A and S6B), alternative macrophages (Figures S2A, S6C and S6D) and fibroblasts (Figure S6E) were not significantly affected. Moreover, TRAM34 did not significantly attenuate macrophage migration or myocardial inflammation (Figure S7). Therefore, reduction of post-MI arrhythmias in our experiments by TRAM34 should be attributed to direct inhibition of KCa3.1 channels, but not attenuation of macrophage polarization or cardiac remodeling.

### Clinical significance

To explore whether mononuclear cells in MI patients correlate with post-MI arrhythmias, we analyzed the monocyte subsets in the peripheral blood samples obtained from MI patients and healthy controls. Our flow cytometry results showed that the percentage of CD14^++^CD16^-^ pro-inflammatory monocytes is significantly higher in patients with post-MI arrhythmias than healthy controls and MI patients without arrhythmias (p<0.001, Figure 1). The pro-inflammatory monocyte subsets are the direct precursors of macrophages in inflammatory tissues 22, 23, and higher levels of peripheral pro-inflammatory monocytes suggest higher levels of macrophage accumulation in MI border zones. Elevation of classical pro-inflammatory monocytes has been demonstrated to be associated with poor cardiac function in MI patients 24. However, whether levels of mononuclear cells in MI patients correlate with post-MI arrhythmias is poorly defined. Our results provided evidences of relationship between higher level of peripheral pro-inflammatory monocytes and higher risk of post-MI arrhythmias. We also performed immunostaining in tissues from both MI patients and MI mice, and both the results demonstrated that macrophages formed gap junction with cardiomyocytes in MI border zones.

### Study limitations

Macrophage specific KCa3.1 deletion mice cannot be applied in our experiments. KCa3.1 is recognized to play crucial roles in activation and proliferation of immune cells including macrophages. Previous studies showed that blocking KCa3.1 inhibited macrophage polarization towards the M1 phenotype 10. In vivo application of TRAM34 for 4 weeks also attenuated MI-induced ventricular remodeling by inhibiting cardiac fibroblasts proliferation 25. Therefore, post-MI macrophage accumulation and fibrosis would be attenuated in macrophage specific KCa3.1 loss-of-function mice, which interferes the determination of the direct effects of KCa3.1 channels. In our study, TRAM34 was applied in vivo for only 3 days, and macrophage polarization and fibrosis were not significantly affected. Moreover, the group sizes of the clinical experiment were limited, as most of the MI patients would receive percutaneous coronary intervention within the time window. More detailed clinical trials with larger group sizes are needed to investigate the roles of mononuclear cells in MI patients. Furthermore, macrophages would polarize into M2 subtype in MI healing stage, and further experiments are needed to investigate the roles of M2 macrophages in the arrhythmias in later stage of MI.

## Conclusions

Macrophages accumulate and polarize into pro-inflammatory subtype in MI border zones, modulate the electrophysiological properties of cardiomyocytes via gap junction and KCa3.1 activation, and predispose the heart to post-MI repolarization heterogeneity and arrhythmias. Macrophages and KCa3.1 ion currents are potential therapeutic targets against post-MI arrhythmias.

## Supplementary Material

Supplementary figures and tables.

## Figures and Tables

**Table 1:** Electrophysiological properties of solitary cardiomyocytes cocultured with macrophages and cardiomyocytes connected with M0 or M1 macrophages.

	APD*10 (ms)	APD50 (ms)	APD90 (ms)	RMP ^†^ (mV)	Cell Capacitance (pF)
Solitary NMVMs ^‡^ cocultured with M0	3.21±0.58	14.49±1.94	52.14±3.81	-66.30±1.08	22.74±1.76
NMVMs connected with M0	2.54±0.48	13.32±1.62	49.19±2.74	-59.06±1.49	35.28±3.07
Solitary NMVMs cocultured with M1	2.90±0.35	17.85±2.86	62.35±3.89	-66.09±0.86	24.97±2.37
NMVMs connected with M1	4.31±0.69	41.80±2.01^§^	139.31±9.20^§^	-56.77±0.90^§^	43.55±4.36^§^

* APD: action potential duration; † RMP: resting membrane potential; ‡ NMVM: neonatal mouse ventricular myocyte, §p<0.01 vs solitary NMVMs cocultured with M1.

## Abbreviations

APD: action potential duration
Cx43: connexin43
DMEM: Dulbecco modified Eagle medium
ECG: electrocardiogram
FBS: fetal bovine serum
LPS: lipopolysaccharide
MI: myocardial infarction
NMVM: neonatal mice ventricular cardiomyocytes
PBMC: peripheral blood mononuclear cell
PVC: premature ventricular contraction
RMP: resting membrane potential
siRNA: small interfering ribonucleic acid
SEM: standard error of mean
SOC: store-operated Ca^2+^
VF: ventricular fibrillation
VT: ventricular tachycardia
