# Supplementary material for: Macrophages facilitate post myocardial infarction arrhythmias: roles of gap junction and KCa3.1
Source: Theranostics. 2019 Aug 14;9(22):6396–411. doi: 10.7150/thno.34801 (PMC6771231; doi:10.7150/thno.34801)
Supplement: Supplementary file 1 — Supplementary figures and tables. [file thnov09p6396s1.pdf]

# Supplementary material

## Macrophages facilitate post myocardial infarction arrhythmias: roles of gap junction and KCa3.1

Yu-Dong Fei [1#]; Qian Wang[1#]; Jian-Wen Hou[1#]; Wei Li[1]; Xing-Xing Cai[1]; Yu-Li Yang[1]; Liu-Hui Zhang[2]; Zhi-Xing Wei[1]; Tai-Zhong Chen[1]; Yue-Peng Wang[1]; Yi-Gang Li[1]*

1 Department of Cardiology, XinHua Hospital Affiliated to Shanghai Jiao Tong University School of Medicine, Shanghai, China

2 Department of Ophthalmology, XinHua Hospital Affiliated to Shanghai Jiao Tong University School of Medicine, Shanghai, China

[#] These authors contributed equally.

* Correspondence:

Dr. Yi-Gang Li, M.D., FACC, FHRS

Department of Cardiology, XinHua Hospital, Shanghai Jiao Tong University School of Medicine

1665 Kongjiang Road, Shanghai, 200092, China

**Table S1**. Characteristics of control and MI patients.

| | Control (n=8) | MI without arrhythmias (n=8) | Post-MI arrhythmias (n=8) |
|---|---|---|---|
| Age (y) | 80.38±3.44 | 61.00±5.20 | 70.88±5.01 |
| Male | 5 | 8 | 5 |
| $CD14^{++}CD16^{-}$ (%) | 61.76±5.00 | 70.18±1.88 | 81.84±0.97* |
| $CD14^{++}CD16^{+}$ (%) | 6.51±1.22 | 6.31±1.38 | 8.31±1.04 |
| $CD14^{+}CD16^{+}$ (%) | 11.64±2.58[#] | 5.64±0.80 | 2.14±0.46 |

* p<0.05 vs controls and MI patients without arrhythmias by Bonferroni post hoc analysis.

[#] p<0.05 vs MI patients without arrhythmias and post-MI arrhythmia patients by Bonferroni post hoc analysis.

**Table S2**. The most differentially expressed genes encoding ion channels by RNA sequencing.

| Gene | Regulation | Fold change | Q value |
|---|---|---|---|
| *Kcnn4* | Up | 63.42 | $1.07\times10^{-24}$ |
| *Orai1* | Up | 2.98 | $2.26\times10^{-4}$ |
| *Orai2* | Up | 5.06 | $2.26\times10^{-7}$ |
| *Kcna5* | Down | -3.18 | $1.81\times10^{-3}$ |
| *Cacna1c* | Down | -5.46 | $2.51\times10^{-5}$ |
| *Scn5a* | Down | -19.31 | $1.12\times10^{-5}$ |

**Table S3**. APDs of solitary cardiomyocytes cocultured with M1 and cardiomyocytes connected with M1 macrophages before and after application of GAP26.

| | APD10 (ms) | APD50 (ms) | APD90 (ms) |
|---|---|---|---|
| solitary NMVMs cocultured with M1 (n=6) | 2.19±0.78 | 21.32±4.26 | 74.22±4.15 |
| solitary NMVMs cocultured with M1 +GAP26 (n=6) | 3.14±0.88 | 20.19±3.95 | 72.54±4.56 |
| NMVMs connected with M1 (n=6) | 4.61±1.17 | 48.62±9.97* | 139.50±10.37* |
| NMVMs connected with M1 + GAP26 (n=6) | 1.89±0.49 | 17.35±4.40# | 79.64±9.59# |

*p<0.05 vs solitary NMVMs cocultured with M1 macrophage.

#p<0.05 vs NMVMs connected with M1 macrophage.

**Table S4**. APDs of solitary cardiomyocytes cocultured with M1 and cardiomyocytes connected with M1 macrophages before and after application of TRAM34.

| | APD10 (ms) | APD50 (ms) | APD90 (ms) |
|---|---|---|---|
| solitary NMVMs cocultured with M1 (n=6) | 2.82±1.14 | 18.62±5.33 | 71.57±4.48 |
| solitary NMVMs cocultured with M1 +TRAM34 (n=6) | 1.91±0.35 | 17.47±2.39 | 67.04±4.07 |
| NMVMs connected with M1 (n=6) | 5.77±1.69 | 51.37±2.60* | 137.70±11.07* |
| NMVMs connected with M1 +TRAM34 (n=6) | 3.06±0.88 | 27.53±6.69[#] | 91.84±8.36[#] |

*p<0.01 vs solitary NMVMs cocultured with M1 macrophage.

[#]p<0.01 vs NMVMs connected with M1 macrophage.

**Table S5**. APDs of solitary cardiomyocytes cocultured with M1 and cardiomyocytes connected with M1 macrophages after application of KCa3.1 siRNA or negative control.

| | APD10 (ms) | APD50 (ms) | APD90 (ms) |
|---|---|---|---|
| solitary NMVMs cocultured with M1 + control (n=5) | 0.74±0.14 | 5.81±2.04 | 53.35±5.69 |
| solitary NMVMs cocultured with M1 +siRNA (n=5) | 1.35±0.64 | 7.01±3.38 | 46.16±3.85 |
| NMVMs connected with M1 + control (n=6) | 3.80±1.04 | 44.41±3.30* | 135.48±10.59* |
| NMVMs connected with M1 +siRNA (n=6) | 3.93±0.96 | 23.71±3.98# | 74.39±6.10# |

*p<0.01 vs solitary NMVMs cocultured with M1 macrophage + control.

#p<0.01 vs NMVMs connected with M1 macrophage + control.

**Table S6**. APDs of solitary cardiomyocytes cocultured with M1 and cardiomyocytes connected with M1 macrophages before and after application of SKF96365.

| | APD10 (ms) | APD50 (ms) | APD90 (ms) |
|---|---|---|---|
| solitary NMVMs cocultured with M1 (n=5) | 1.81±0.54 | 10.61±3.50 | 57.84±7.92 |
| solitary NMVMs connected with M0 +SKF96365 (n=5) | 2.09±0.86 | 15.15±6.97 | 57.34±7.64 |
| NMVMs cocultured with M1 (n=5) | 6.51±1.97 | 44.11±3.95* | 136.01±13.94* |
| NMVMs connected with M1 +SKF96365 (n=5) | 1.45±0.50 | 14.82±7.46# | 74.77±7.71# |

*p<0.01 vs solitary NMVMs cocultured with M1 macrophage.

#p<0.01 vs NMVMs connected with M1 macrophage.

**Table S7**. Heart rates and QTc durations of MI and sham operated mice treated with TRAM34 or vehicle.

| | Heart rate (bpm) | QTc (ms) |
|---|---|---|
| Sham + vehicle (n=5) | 579.74±14.98 | 56.41±2.66 |
| Sham + TRAM34 (n=6) | 545.28±10.34 | 58.93±1.20 |
| MI + vehicle (n=6) | 634.22±13.22 | 69.27±2.11 |
| MI + TRAM34 (n=5) | 601.82±13.99 | 63.03±0.90* |

*p<0.05 vs MI+vehicle.

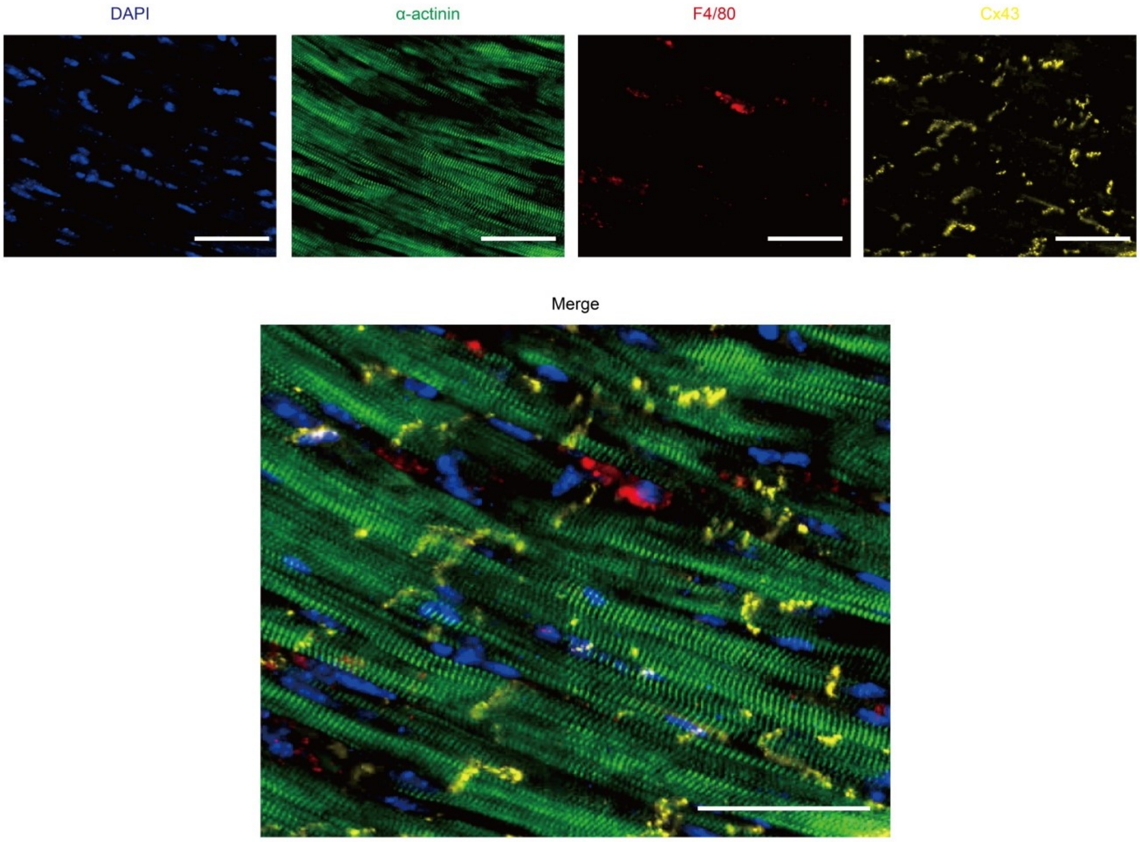


**Figure S1.** Gap junctions were mainly distributed between cardiomyocytes in sham operated mouse ventricles. Triple immunofluorescence (cardiomyocytes = green, macrophages = red, Cx43 = yellow) of sham mouse ventricle showed that Cx43 was mainly detected between cardiomyocytes. Scale bars indicate 50 μm.

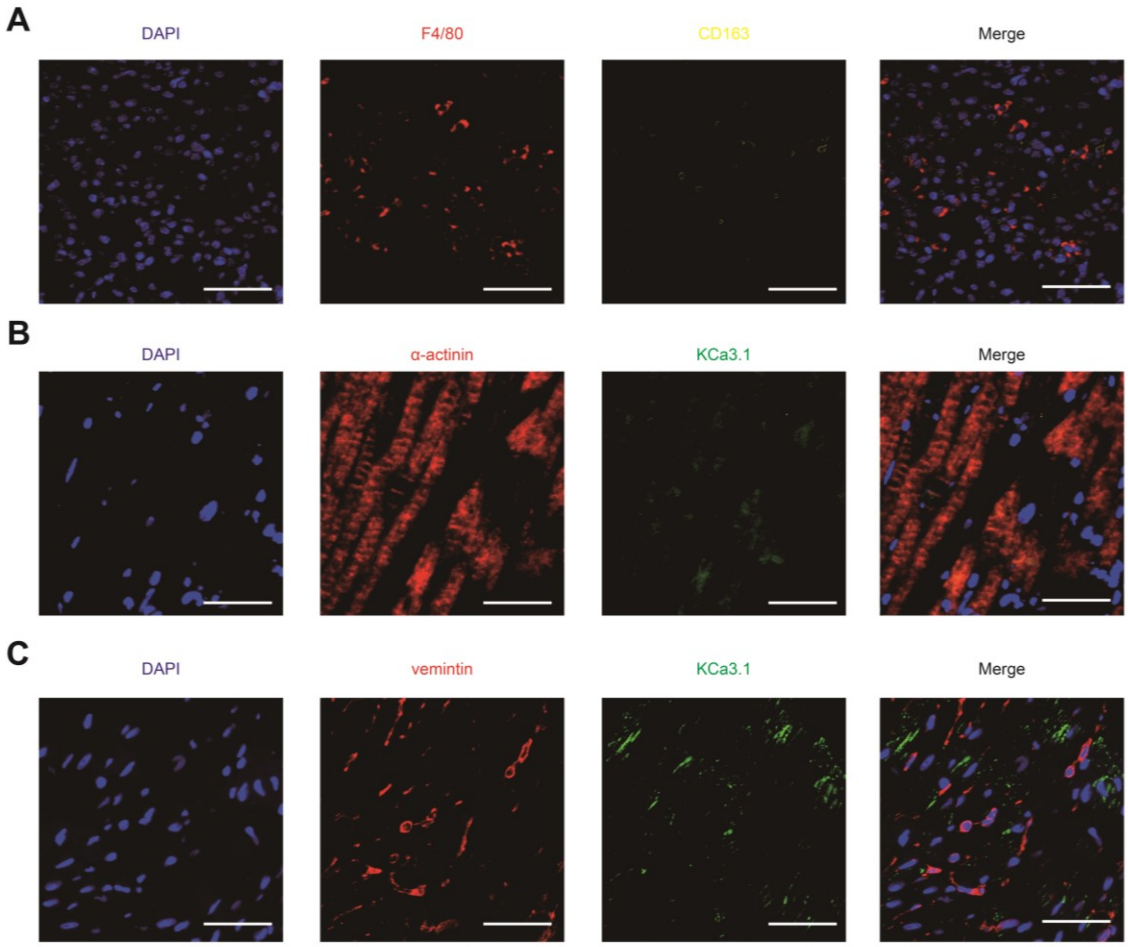


**Figure S2**. Alternative macrophages and KCa3.1 expression were analyzed by immunofluorescence in MI border zones at 3 days post-MI. (A). Double immunofluorescence of F4/80 (red) and CD163 (yellow) in MI border zone of MI mice. (B) Double immunofluorescence of α- actinin (red) and KCa3.1 (green) in MI border zone of MI mice. (C) Double immunofluorescence of vimentin (red) and KCa3.1 (green) in MI border zone of MI mice. Scale bars indicate 50 μm.

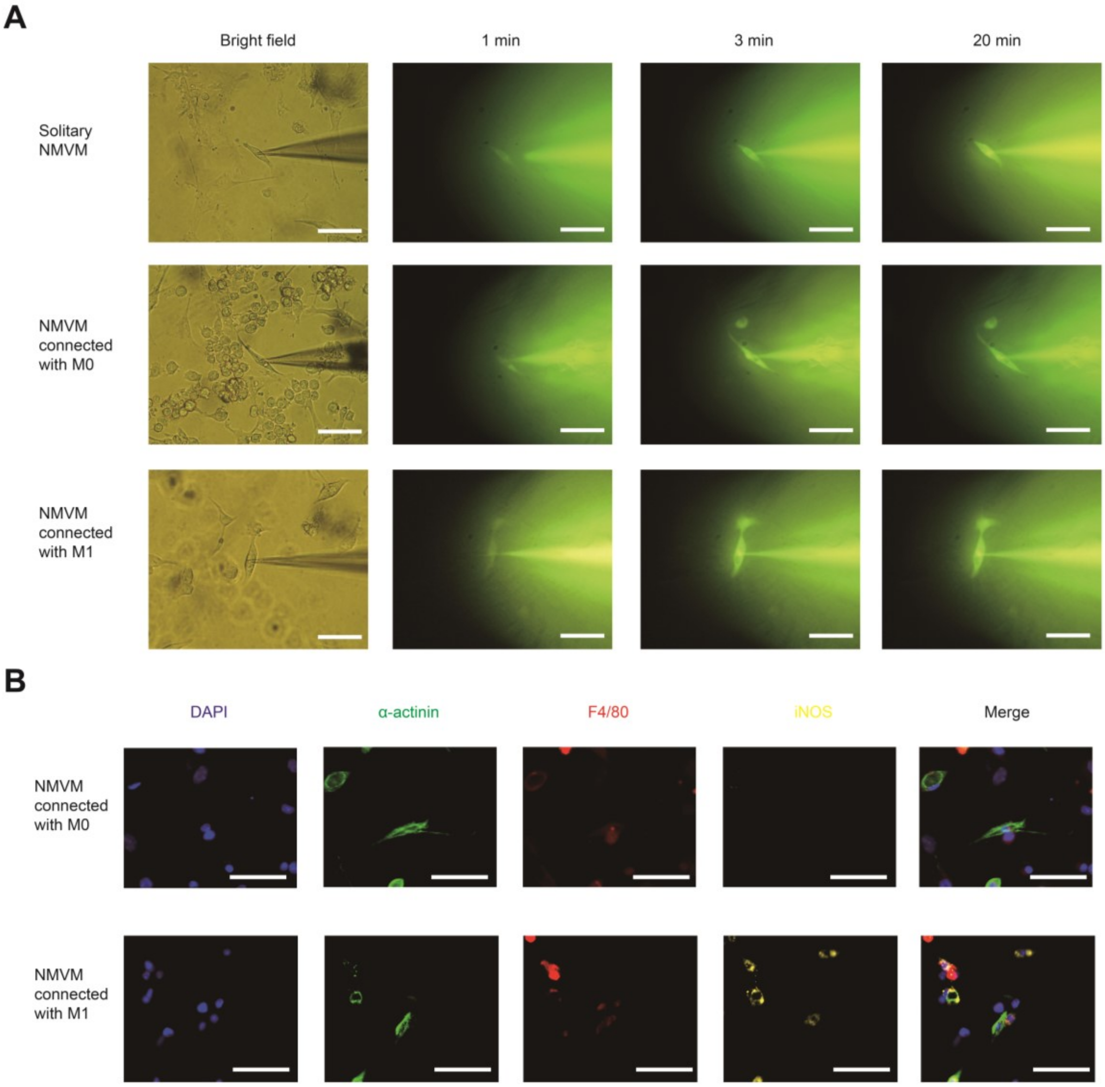


**Figure S3**. Solitary NMVMs and NMVMs connected with macrophages were identified. (A). Photographs captured under bright field and at 1st minute, 3rd minute and 20st minute after cell membrane rupture under fluorescence during patch clamping. Solitary NMVMs cocultured with macrophages were confirmed both by no adjacent cells on bright field and no lucifer yellow transfer under fluorescence. Gap junction formation between macrophages and cardiomyocytes were indicated by lucifer yellow transfer. (B). Representative results of triple immunofluorescence of α-actinin (green), F4/80 (red) and iNOS (yellow) of cardiomyocyte connected with M0 ($F4/80^+$ $iNOS^-$) or M1 ($F4/80^+$ $iNOS^+$) macrophage. Scale bars indicate 50 μm.

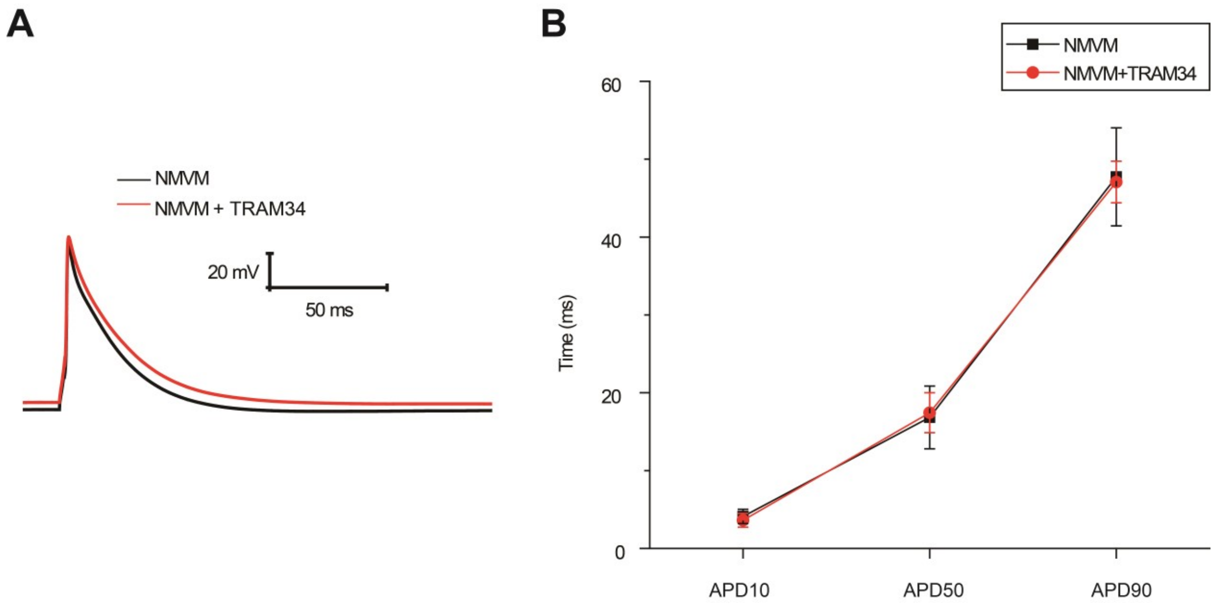


**Figure S4**. TRAM34 did not exert significant effect on action potentials of cardiomyocyte. (A). Representative action potential curves of NMVMs before and after TRAM34 treatment. (B). Statistical summary of action potential durations of NMVMs before and after TRAM34 treatment (n=5).

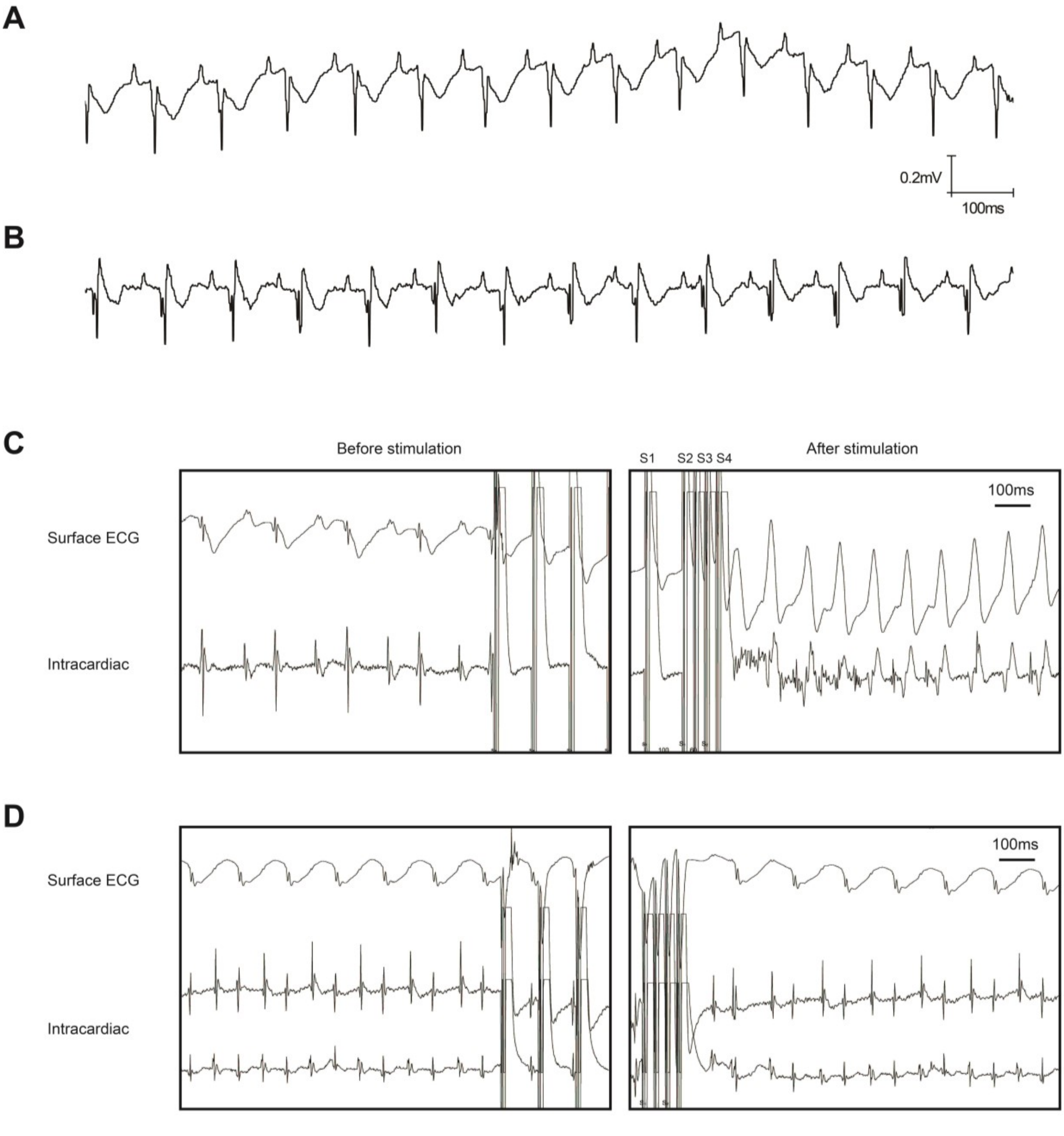


**Figure S5**. KCa3.1 inhibition effectively attenuated QT prolongation and extra-stimulus induced post-MI arrhythmias at 3 days post-MI. (A-B). Representative ambulatory ECG recordings of MI mice treated with vehicle (A) or TRAM34 (B). (C-D). Representative surface ECGs and intracardiac images during intracardiac extra-stimulus experiment of MI mice treated with vehicle (C) or TRAM34 (D).

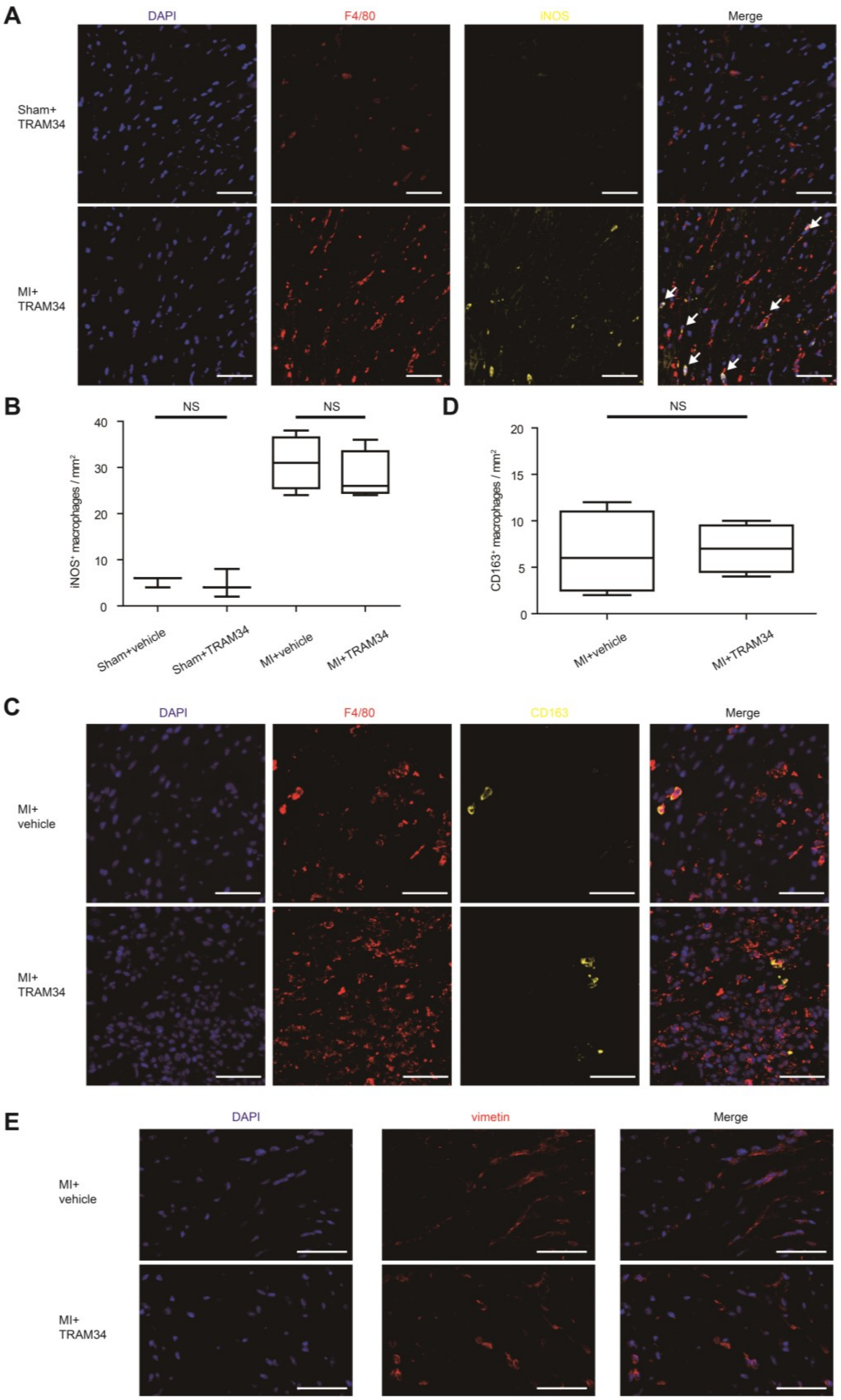


**Figure S6**. TRAM34 did not change the levels of pro-inflammatory and alternative macrophages in MI

border zones 3 days post-MI. (A). Double immunofluorescence of F4/80 (red) and iNOS (yellow) in MI border zone of MI or sham operated mice treated with TRAM34. Results of MI or sham operated mice treated with vehicle were shown in Figure 2. (B). Statistical summary of pro-inflammatory macrophage counts in sham (n=3 in each group) and MI (n=4 in each group) mouse ventricles. (C). Double immunofluorescence of F4/80 (red) and CD163 (yellow) in MI border zone of MI mice treated with TRAM34. (D). Statistical summary of M2 macrophage density (n=4 in each group). (E). Immunofluorescence of vimetin in MI border zone of MI mice treated with vehicle or TRAM34. Scale bars indicate 50 μm. NS, not significant.

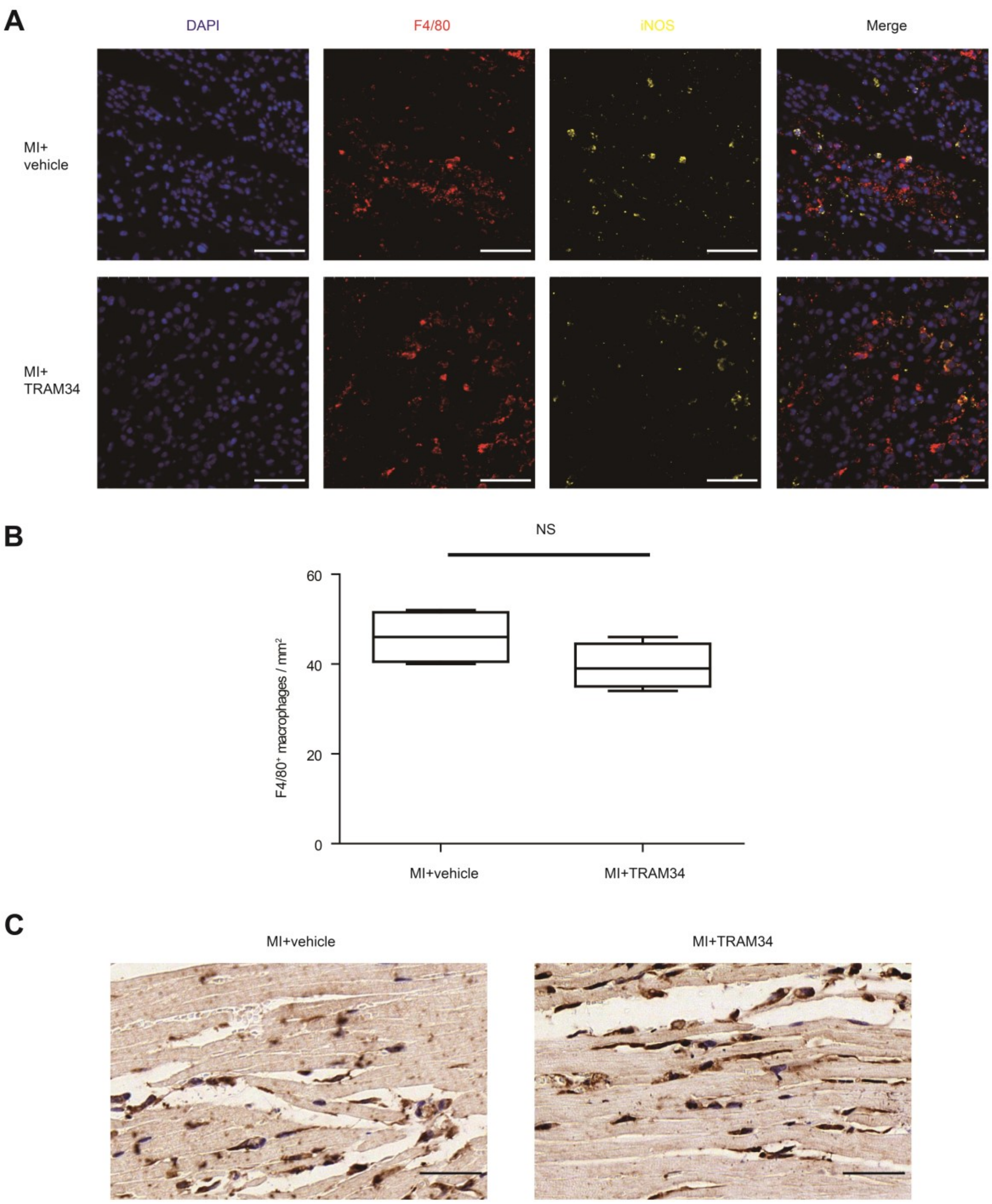


**Figure S7.** TRAM34 did not affect macrophage migration or myocardial inflammation in infarct zones at 3 days post-MI. (A). Double immunofluorescence of F4/80 (red) and iNOS (yellow) in infarct zones of MI mice treated with vehicle or TRAM34. (B). Statistical summary of macrophage density (n=4 in each group). (C). Immunohistochemical analysis of TNF-α in infarct zones of MI mice treated with vehicle or TRAM34. Scale bars indicate 50 μm. NS, not significant.
